# Quantitative hypoxia mapping using a self-calibrated activatable nanoprobe

**DOI:** 10.1186/s12951-022-01341-9

**Published:** 2022-03-18

**Authors:** Xin Feng, Yuhao Li, Shiyuan Zhang, Changjian Li, Jie Tian

**Affiliations:** 1 CAS Key Laboratory of Molecular Imaging, Beijing Key Laboratory of Molecular Imaging, the State Key Laboratory of Management and Control for Complex Systems, Institute of Automation, Chinese Academy of Sciences, Beijing, 100190 China; 2 School of Artificial Intelligence, University of Chinese Academy of Science, Beijing, 100080 China; 3 Institute of Bismuth Science and School of Materials and Chemistry, University of Shanghai for Science and Technology, Shanghai, 200093 China; 4 Beijing Advanced Innovation Center for Big Data-Based Precision Medicine, School of Medicine and Engineering, Beihang University, Beijing, 100191 China; 5 Engineering Research Center of Molecular and Neuro Imaging of Ministry of Education, School of Life Science and Technology, Xidian University, Xi’an, Shaanxi, 710126 China; 6 Key Laboratory of Big Data-Based Precision Medicine (Beihang University), Ministry of Industry and Information Technology, Beijing, 100191 China

**Keywords:** Hypoxia, Ratiometric imaging, Quantitative, Breast cancer

## Abstract

Hypoxia is a distinguished hallmark of the tumor microenvironment. Hypoxic signaling affects multiple gene expressions, resulting in tumor invasion and metastasis. Quantification of hypoxic status although challenging, can be useful for monitoring tumor development and aggressiveness. However, hypoxia-independent factors such as nonspecific binding and heterogenous probe delivery considerably influence the probe signal thereby disenabling reliable quantitative imaging in vivo. In this study, we designed a self-calibrated activatable nanoprobe Cy7-1/PG5-Cy5@LWHA that specifically detects nitroreductase activity upregulated in hypoxic tumor cells. Dual fluorescence emission of the nanoprobe enables ratiometric calibration and eliminates the target-independent interference. In orthotopic and metastatic breast cancer mouse models, Cy7-1/PG5-Cy5@LWHA demonstrated remarkable hypoxia sensing capability in vivo. Moreover, ratiometric processing provided quantitative hypoxia assessment at different tumor developmental stages and facilitated tumor burden assessment in the metastatic lymph nodes. Therefore, our study demonstrates that ratiometric imaging of Cy7-1/PG5-Cy5@LWHA can be a prospective noninvasive tool to quantitatively monitor tumor hypoxia, which would be beneficial for investigating the fundamental role of hypoxia in tumor progression and for evaluating response to novel anti-hypoxia therapeutics. Furthermore, successful detection of metastatic lymph nodes with the proposed imaging approach illustrates its potential clinical application in assessing lymph node status during surgery.

**Graphical Abstract:**

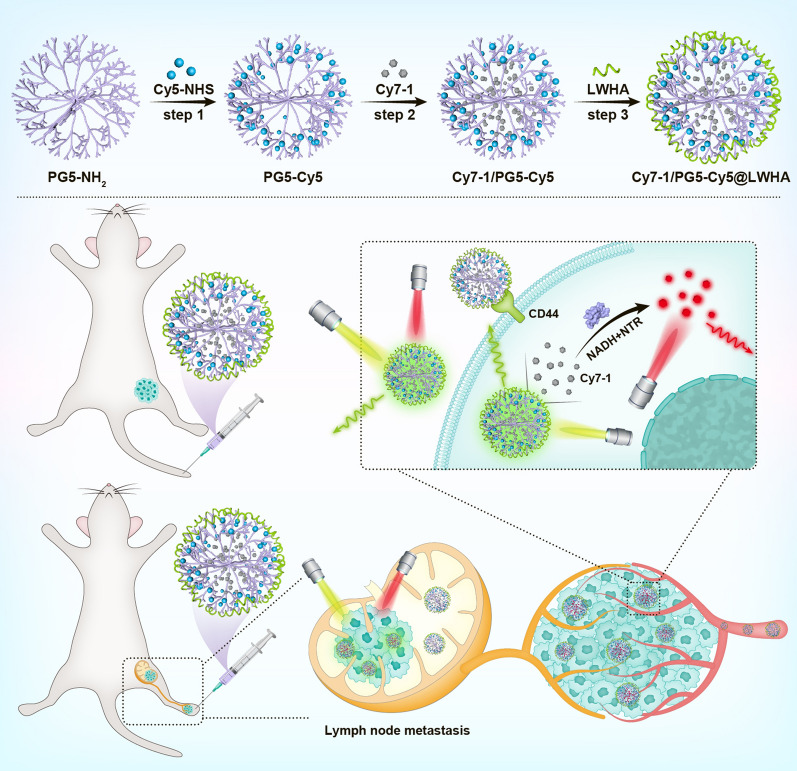

**Supplementary Information:**

The online version contains supplementary material available at 10.1186/s12951-022-01341-9.

## Introduction

Hypoxia, resulting from the rapid proliferation of cancer cells and poor tumor vasculature, is one of the most pervasive features of solid tumors [1]. In order to adapt to the oxygen-deprived microenvironment, hypoxia-inducible transcription factor (HIF-1) is activated in cancer cells, which subsequently upregulates genes associated with metabolic alteration, cell migration, metastasis, and treatment resistance [2–5]. Therefore, hypoxia is often identified as an indicator of tumor aggressiveness and poor prognosis. Recently, it has also been reported that metastatic tumors exhibit the same hypoxia signatures as the primary tumor [6, 7]. In this context, hypoxia imaging is beneficial for evaluating tumor malignancy and can be helpful for identifying tumor metastasis as well.

Over the past decades, nitroreductase (NTR) has been identified as a biomarker for hypoxia [8–10]. It has been reported that NTR activity is elevated in hypoxic tumors [8], ischemic myocardial tissue [9], and bacterial infections [10]. NTR catalyzes the reduction of nitro compounds to amines in the presence of NAD(P)H [11]. Based on this reaction, multiple NTR-activatable fluorescence probes have been designed for tumor hypoxia imaging, most of them are based on quinones or nitroaromatic compounds [12–17]. Activatable probes have greatly improved the imaging specificity since fluorescence signal is only turned on in hypoxic tumor cells. However, hypoxia assessment in vivo remains to be challenging, since vascular abnormalities associated with hypoxia [18, 19] often affect probe delivery, leading to heterogeneous local probe concentration. As a result, in vivo probe signal cannot reliably reflect the NTR activity, thus hinders the accurate hypoxia quantification.

Herein, we designed a self-calibrated NTR activatable nanoprobe Cy7-1/PG5-Cy5@LWHA that enables accurate hypoxia imaging. Activatable fluorescent reporter Cy7-1 was used to sense the NTR activity [15], and an “always-on” Cy5 fluorophore was employed as an internal reference to account for heterogeneity in probe delivery. Two fluorophores were incorporated together through the poly(amidoamine) dendrimer (5^th^ generation, PG5), and the nanoconjugate was modified with low molecular weight hyaluronic acid (LWHA, molecular weight 5 kDa) to enable cell internalization through CD44. Our results demonstrate that ratiometric imaging of Cy7-1/PG5-Cy5@LWHA enabled quantitative assessment of hypoxia in vivo in orthotopic and metastatic breast cancer models. In vivo imaging results agree with the findings of ex vivo semi-quantitative techniques including immunofluorescence and qPCR. The present study delivers a noninvasive tool to quantify the extent of tumor hypoxia, which would facilitate the study of tumor biology and the development of anti-hypoxia strategies. The successful detection of metastatic lymph node (LN) using this approach further suggests its potential use in LN assessment during surgery.

## Results

### Design, synthesis, and characterization of the self-calibrated activatable nanoprobe

**Scheme 1:**
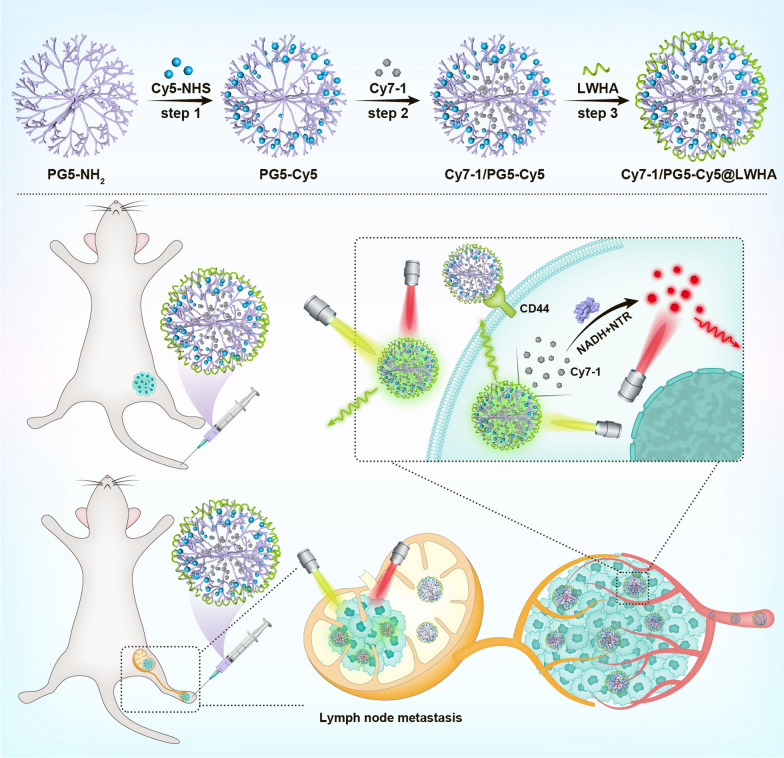
Synthesis route, structure and function of the self-calibrated activatable nanoprobe Cy7-1/PG5-Cy5@LWHA. Top: synthesis route and structure of Cy7-1/PG5-Cy5@LWHA. Bottom: In vivo imaging of Cy7-1/PG5-Cy5@LWHA allowed for quantitative hypoxia imaging in orthotopic and LN metastatic breast cancer models. “Always-on” Cy5 in the nanoprobe is used to monitor probe accumulation, and Cy7-1 in the nanoprobe is used to sense NTR activity in the hypoxic tumor cells. The nanoprobe was administered through tail-vein injection in orthotopic tumor model and through intratumoral injection in LN metastatic models

**Fig. 1:**
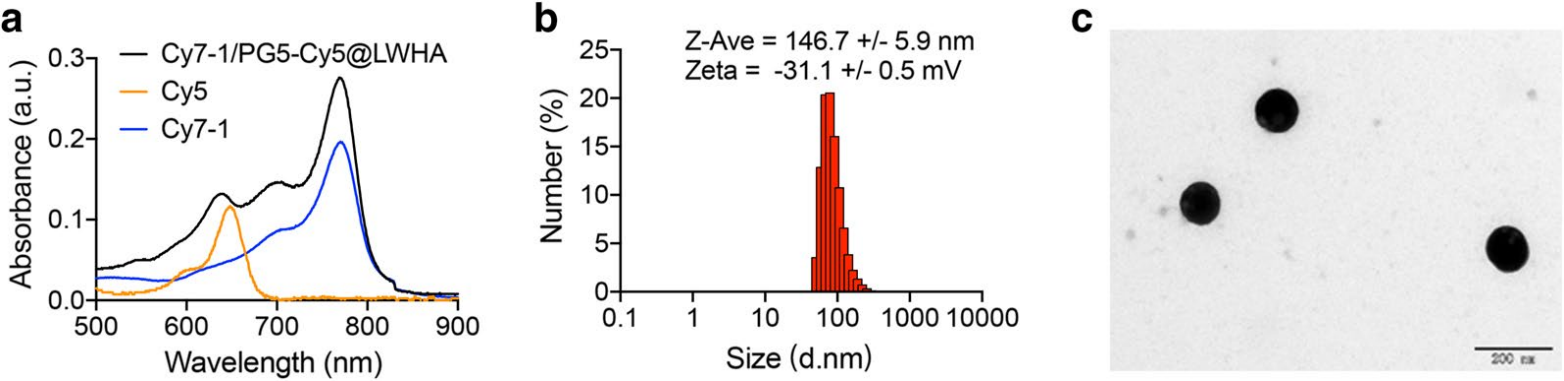
Characterization of Cy7-1/PG5-Cy5@LWHA. **a** UV–vis absorption spectra of Cy5 (orange), Cy7-1 (blue), Cy7-1/PG5-Cy5@LWHA (black). **b** Hydrodynamic size and zeta potential of Cy7-1/PG5-Cy5@LWHA nanoprobe. **c** TEM image of the nanoprobe, scale bar: 200 nm

Hypoxia-activatable NIR dye Cy7-1, an “always-on” fluorophore Cy5, and LWHA were incorporated together using PG5 to form a self-calibrated activatable nanoprobe Cy7-1/PG5-Cy5@LWHA. PG5 was selected as the nanocarrier because of its consistent nanometer size, globular shape, and polyfunctional dendrimer surface [20]. Synthesis route, structure and function of the nanoprobe are illustrated in Scheme 1. Briefly, Cy5-NHS was covalently conjugated to the peripheral amino groups on PG5, with a labeling rate of 2% (PG5-Cy5). After purification, hydrophobic Cy7-1 was encapsulated in the internal cavities of PG5-Cy5 at a loading rate of 8% (Cy7-1/PG5-Cy5). The CD44 targeting group LWHA [21] was coated on the surface of Cy7-1/PG5-Cy5 to form Cy7-1/PG5-Cy5@LWHA. After purification, Cy7-1/PG5-Cy5@LWHA was characterized using Fourier transform infrared spectroscopy (Figure S1). The absorption spectra of Cy7-1, Cy5, and Cy7-1/PG5-Cy5@LWHA are presented in Fig. 1a. Cy7-1/PG5-Cy5@LWHA (black curve) showed two distinct absorption peaks around 620 nm and 770 nm, indicating the successful labeling of Cy5 and encapsulation of Cy7-1, respectively. Prior to LWHA modification, the zeta potential of Cy7-1/PG5-Cy5 was approximately + 37.4 mV, which could lead to cell toxicity and low retention time in circulation [22]. Negatively charged LWHA neutralized the cationic surface charge and shifted the zeta potential to -31.1 mV (Fig. 1b). The hydrodynamic size of Cy7-1/PG5-Cy5@LWHA was 146.7 nm. Transmission electron microscopy (TEM) revealed that the nanoprobes were well-separated spheres with a size of approximately 108 nm (Fig. 1c).

### NTR response of the nanoprobe

**Fig. 2:**
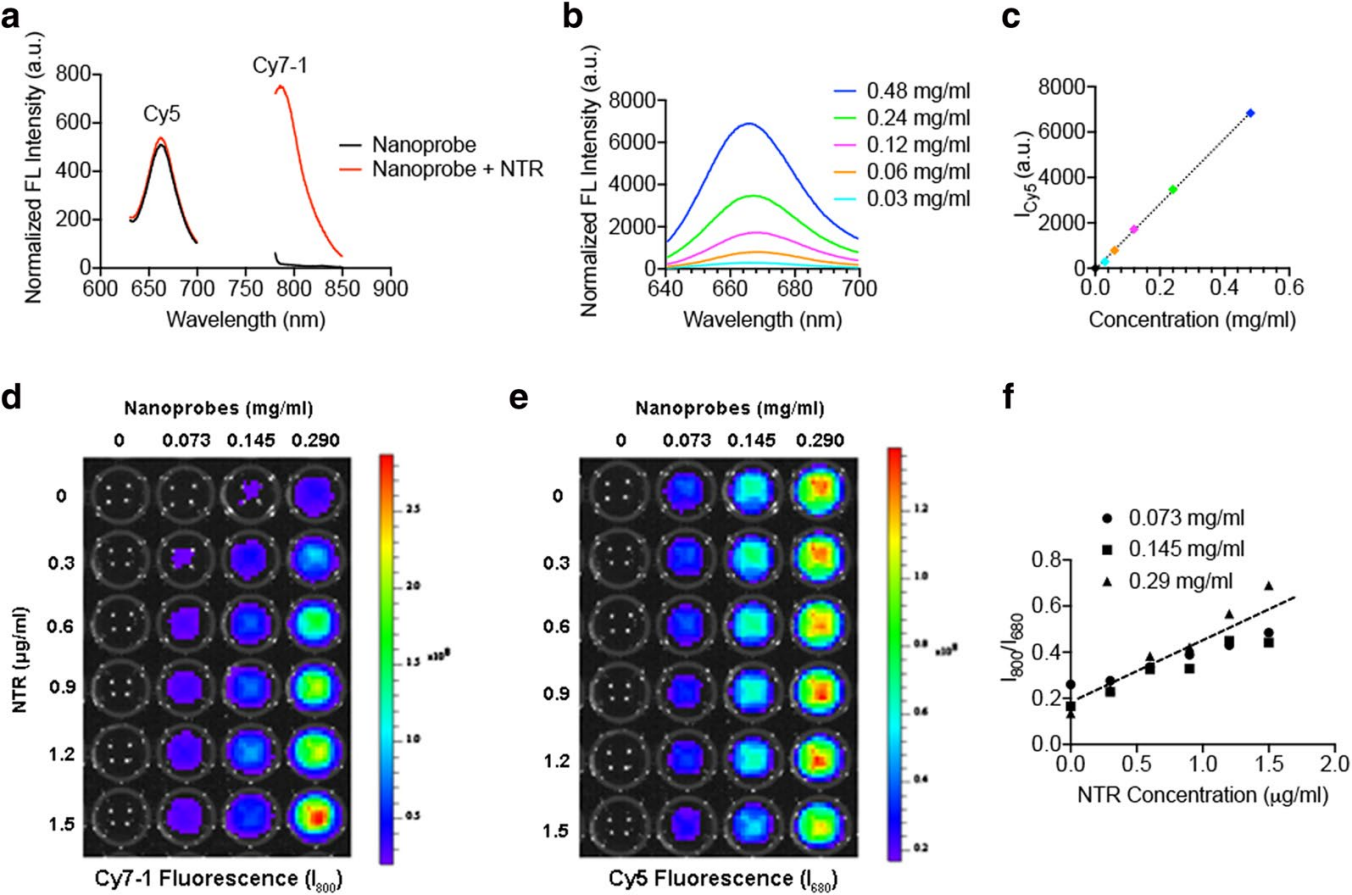
NTR response and ratiometric imaging of the Cy7-1/PG5-Cy5@LWHA nanoprobe. **a** Fluorescence spectra of the nanoprobe with (red) and without (black) the presence of NTR; emission peak at 666 nm corresponds to Cy5 fluorescence and emission peak at 785 nm corresponds to the activated Cy7-1 fluorescence. **b** Cy5 fluorescence emission at different nanoprobe concentrations. **c** Linear relationship between Cy5 fluorescence emission and the nanoprobe concentration. **d** Cy7-1 fluorescence emission (I_800_) at different nanoprobe concentrations (left to right: 0, 0.073 mg/ml, 0.145 mg/ml, 0.290 mg/ml) and NTR levels (top to bottom: 0, 0.3 ${\mu g}/{\text{ml}}$, 0.6 ${\mu g}/{\text{ml}}$, 0.9 ${\mu g}/{\text{ml}}$, 1.2 ${\mu g}/{\text{ml}}$, 1.5 ${\mu g}/{\text{ml}}$). **e** Cy5 fluorescence emission (I_680_) at different nanoprobe concentrations (left to right: 0, 0.073 mg/ml, 0.145 mg/ml, 0.290 mg/ml) and NTR levels (top to bottom: 0, 0.3 ${\mu g}/{\text{ml}}$, 0.6 ${\mu g}/{\text{ml}}$, 0.9 ${\mu g}/{\text{ml}}$, 1.2 ${\mu g}/{\text{ml}}$, 1.5 ${\mu g}/{\text{ml}}$). **f** I_800_/I_680_ ratiometric analysis eliminated the concentration effect and presented linear relationship with NTR concentration

The fluorescence emission spectra of Cy7-1/PG5-Cy5@LWHA are presented in Fig. 2a. Under normal physiological conditions (37 ºC, pH 7.4), the emission peak was located around 666 nm when excited at 635 nm. No obvious emission was detected when excited at 769 nm. To investigate the NTR response of the nanoprobe in vitro, 0.5 μg/ml NTR and 0.35 mg/ml NADH in Tris buffer were used [15]. After incubating with the NTR/NADH solution for 5 min at 37 ºC, Cy7-1/PG5-Cy5@LWHA exhibited a distinct emission peak around 785 nm when excited at 769 nm, indicating that Cy7-1 was reduced to fluorescent Cy7. The fluorescence intensity at 785 nm was 41-fold that measured without NTR (Fig. 2a). To explore whether encapsulation of Cy7-1 could affect its NTR sensitivity, free Cy7-1 was incubated with the above-mentioned NTR/NADH solution under the same conditions. Spectroscopy measurements showed that the fluorescence intensity of free Cy7-1 increased 51-fold after incubation (Figure S2). Thus, encapsulation of Cy7-1 slightly reduced NTR sensitivity, yet the nanoprobe still exhibited sufficient signal enhancement in response to NTR. Conversely, Cy5 fluorescence remained unchanged after the addition of NTR (Fig. 2a), and was linearly correlated with the nanoprobe concentration (Fig. 2b, c). Therefore, Cy5 fluorescence was used as an internal reference to eliminate the effect of local probe concentration on NTR quantification.

To validate our ratiometric approach to quantify NTR, different concentrations of Cy7-1/PG5-Cy5@LWHA were incubated with NTR solutions ranging from 0 to 1.5 μg/ml in a 96-well plate and imaged with the IVIS imaging system. As shown in Fig. 2d, even though Cy7-1 fluorescence (I_800_) increased with NTR concentration (top to bottom), the nanoprobe concentration also affected the signal intensity (left to right). This mimics the in vivo situation where the nanoprobe concentration in the tumor region is heterogeneous due to the differences in vasculature, interstitial pressure, and other physiological factors that may affect probe delivery. The signal enhancement of I_800_ is a combined result of NTR activity and probe accumulation. Thus, I_800_ alone cannot reliably reflect the NTR level. As shown in Fig. 2e, fluorescence of the internal reference Cy5 (I_680_) was unaffected by NTR (top to bottom), directly reflecting the nanoprobe concentration. After ratiometric calibration, the signal ratio (I_800_/I_680_) showed a good linear relationship with the NTR concentration, and was independent of the nanoprobe concentration (Fig. 2f). Thus, ratiometric calibration of the nanoprobe eliminated the concentration effect and demonstrated an outstanding capability for NTR quantification.

### Cellular uptake of the nanoprobe

**Fig. 3:**
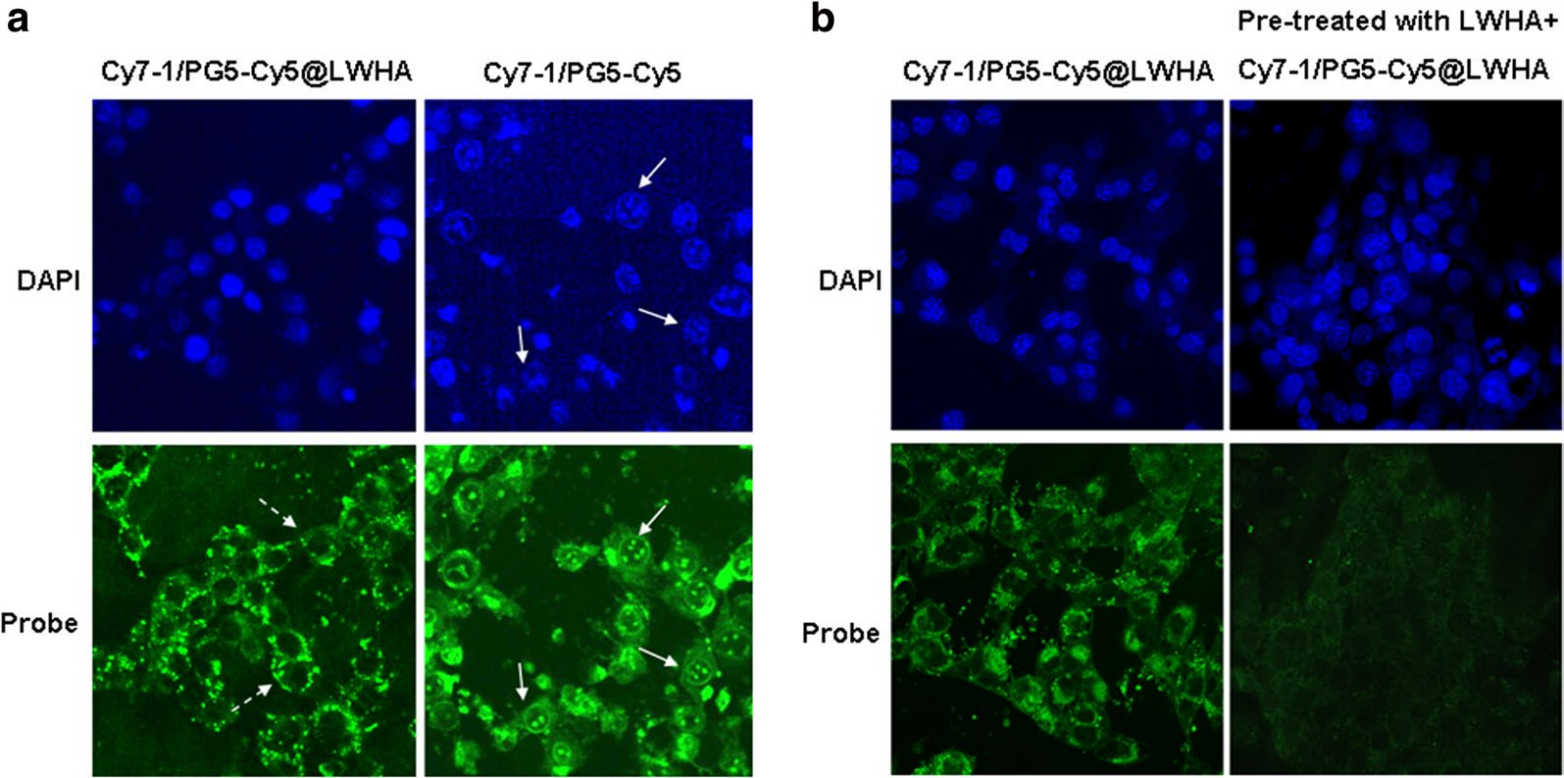
Uptake of Cy7-1/PG5-Cy5@LWHA in 4T1 breast cancer cells. **a** Comparison of the cellular uptake of Cy7-1/PG5-Cy5@LWHA (left) and Cy7-1/PG5-Cy5 (right). **b** Uptake of Cy7-1/PG5-Cy5@LWHA in 4T1 cells with (right) and without (left) pre-treated LWHA. Blue: DAPI staining of the nucleus, green: “always-on” Cy5 fluorescence of the nanoprobe; solid arrows: cell nucleus; dashed arrows: cytoplasm

Previous studies have shown that elevated NTR activity is a distinguished feature of hypoxic tumor microenvironment, and is found inside hypoxic cancer cells [23]. Thus, to allow efficient hypoxia imaging, the nanoprobe needs to enter cancer cells to react with NTR. Previous studies have shown that LWHA can efficiently target CD44 receptor overexpressed on cancer cells and can be internalized via CD44-mediated endocytosis [21]. To explore the cellular uptake pattern of Cy7-1/PG5-Cy5@LWHA, CD44^+^ 4T1 cells were incubated with the nanoprobe (0.05 mg/ml) for 4 h and fluorescence was subsequently observed with a confocal microscope. To investigate the effect of LWHA modification, cells were also incubated with Cy7-1/PG5-Cy5 (0.05 mg/ml) and imaged under the same conditions. As shown in Fig. 3a, both Cy7-1/PG5-Cy5@LWHA and Cy7-1/PG5-Cy5 were taken up by 4T1 cells. Cy7-1/PG5-Cy5@LWHA accumulated in the cell cytoplasm (white dashed arrows), while Cy7-1/PG5-Cy5 entered the cell nucleus (white solid arrows). The different intracellular distributions of the two nanoprobes could be explained by their opposing surface charges and different internalization mechanisms. Cationic Cy7-1/PG5-Cy5 entered the cell and nucleus via nonspecific electrostatic interactions, as reported for other cationic probes. On the other hand, anionic Cy7-1/PG5-Cy5@LWHA entered the cells through CD44 receptors and remained within the cytoplasm upon internalization. Although both nanoprobes were able to get into the cancer cells, it has been reported that cationic probes are prone to cytotoxicity. Therefore, we performed an MTT assay to further investigate the biocompatibility of the two nanoprobes. As shown in Additional file 3: Figure S3, Cy7-1/PG5-Cy5@LWHA exhibited good biocompatibility, while Cy7-1/PG5-Cy5 showed significant cytotoxicity.

To further confirm the internalization mechanism of Cy7-1/PG5-Cy5@LWHA, 4T1 cells were incubated with free LWHA to block CD44 and then incubated with Cy7-1/PG5-Cy5@LWHA. As presented in Fig. 3b, cellular uptake of Cy7-1/PG5-Cy5@LWHA significantly decreased after CD44 receptors were blocked. Overall, our results demonstrated that the Cy7-1/PG5-Cy5@LWHA nanoprobe entered CD44^+^ breast cancer cells through CD44-mediated endocytosis, and later localized to the endolysosomal compartments in the cytoplasm.

### Hypoxia imaging in vitro

**Fig. 4:**
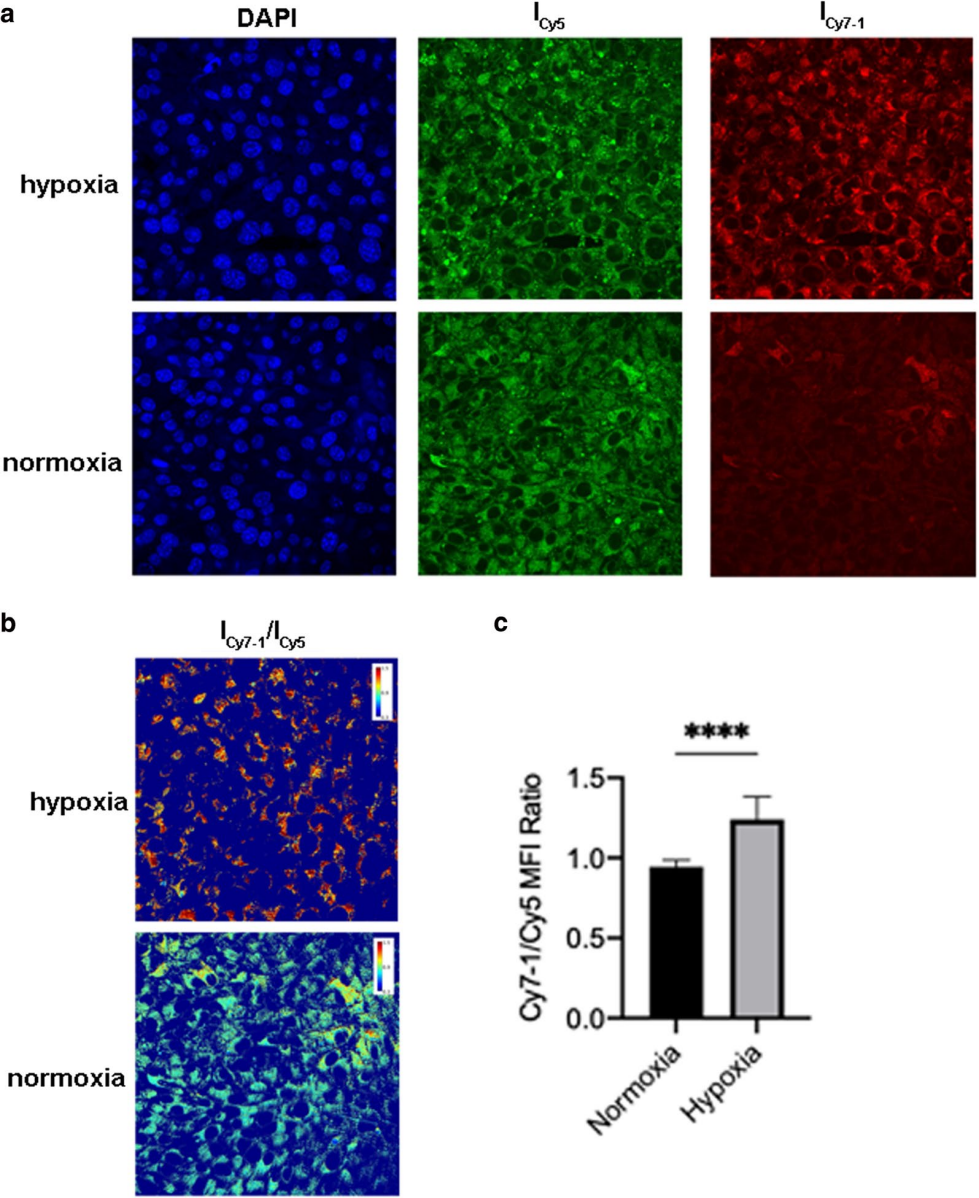
Quantitative hypoxia imaging in vitro. **a** Fluorescence images of hypoxic and normoxic 4T1 cells incubated with Cy7-1/PG5-Cy5@LWHA; blue: DAPI staining of nucleus, green: “always-on” Cy5 fluorescence of the nanoprobe, red: activatable Cy7-1 fluorescence of the nanoprobe. **b** Pseudo-color I_Cy7-1_/I_Cy5_ ratio images of hypoxic and normoxic 4T1 cells incubated with Cy7-1/PG5-Cy5@LWHA. **c** Quantitative analysis of I_Cy7-1_/I_Cy5_ ratio in hypoxic and normoxic 4T1 cells, error bars, mean $\pm$ s.d. (n = 3), *p* < 0.0001

To test the performance of Cy7-1/PG5-Cy5@LWHA with respect to imaging hypoxia in living-cell assays, cobalt chloride (CoCl_2_) was used to induce hypoxia in 4T1 cells [24]. Hypoxic and normoxic cells were incubated with 0.05 mg/ml Cy7-1/PG5-Cy5@LWHA for 4 h and imaged under a confocal microscope. As shown in Fig. 4a, the Cy5 fluorescence was comparable between hypoxic and control cells, indicating that hypoxia did not affect the cellular uptake or intracellular localization of the nanoprobe. In contrast, Cy7-1 fluorescence intensity remarkably increased in hypoxic cells, suggesting the successful activation of the nanoprobe by NTR. We believe that the nanoprobes first underwent conformational change in the low pH environment in lysosomes [25]. Cy7-1 was then released and reacted with NTR. The pseudo color images in Fig. 4b showed notably higher I_Cy7-1_/I_Cy5_ ratio in the cytoplasm of CoCl_2_-treated cells, demonstrating the sensitivity of the ratiometric approach for detecting hypoxia. Quantitative analysis of three independent batches of cells confirmed that the I_Cy7-1_/I_Cy5_ ratio was significantly higher in hypoxic cells than in normoxic cells (*p* < 0.0001).

### In vivo hypoxia imaging in orthotopic 4T1 tumor model

**Fig. 5:**
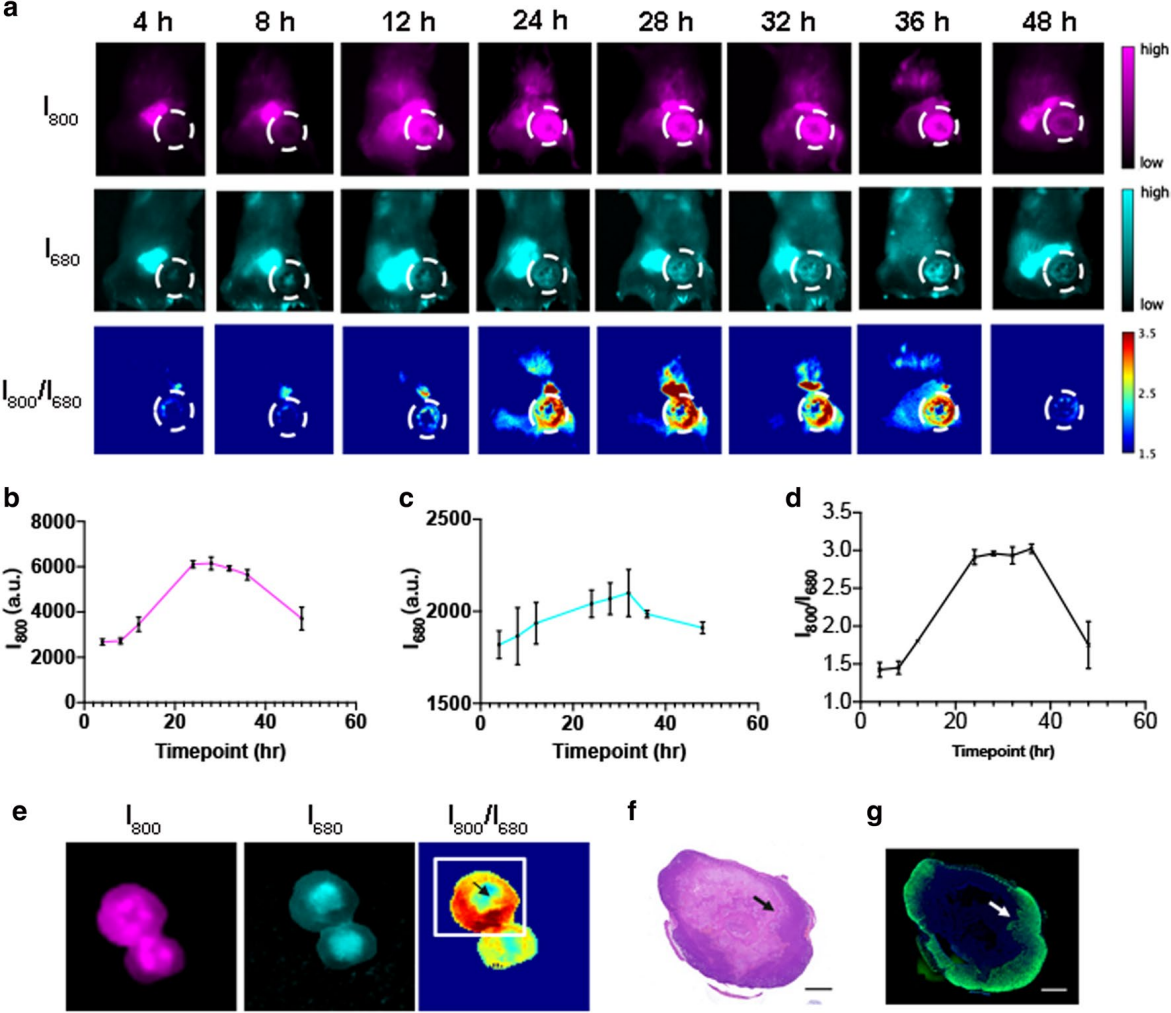
Hypoxia imaging of mice bearing 4T1 orthotopic xenografts. **a** Fluorescence images of Cy7-1 channel (I_800_), Cy5 channel (I_680_) and ratio images (I_800_/I_680_) acquired at each imaging time point after intravenously injecting the nanoprobe (5 mg/kg). **b** Quantitative assessment of the mean fluorescence intensity I_800_ at each time point, error bars, mean $\pm$ s.d. (n = 5). **c** Quantitative assessment of the mean fluorescence intensity I_680_ at each time point, error bars, mean $\pm$ s.d. (n = 5). **d** Quantitative assessment of mean I_800_/I_680_ ratio at each time point, error bars, mean $\pm$ s.d. (n = 5). **e** Representative fluorescence and fluorescence ratio images of the excised tumor tissue. **f** H&E of the tumor tissue outlined by the box in **e**. **g** HIF-1 $\alpha$ staining of the tumor tissue outlined by the box in e. Solid arrows: hypoxic regions

To investigate the feasibility of using Cy7-1/PG5-Cy5@LWHA to quantitatively assess tumor hypoxia in vivo, a mouse model bearing orthotopic 4T1 tumors was established. Tumors were allowed to grow for 4 weeks after inoculation to develop hypoxic conditions. For in vivo imaging, 200 µl Cy7-1/PG5-Cy5@LWHA (1 mg/ml) was administered intravenously and mice were imaged at 4, 8, 12, 24, 28, 32, 36, and 48 h post injection. Fluorescence images of Cy7-1 (I_800_), Cy5 (I_680_), and ratio images (I_800_/I_680_) of a representative case are shown in Fig. 5a. Signals were detected in the tumor region (white dashed circle) starting from 12 h post injection, demonstrating the long circulation time of the nanoprobe and its gradual accumulation in the tumor area. Ratio images taken between 24 and 36 h distinctively outlined the tumor, indicating the successful activation of the nanoprobe. Quantitative analysis of five mice confirmed that both I_800_ and I_680_ started to increase at 12 h, reached a plateau at 24 h, and remained stable up to 36 h, before diminishing at 48 h post injection (Fig. 5b, c). Similarly, the I_800_/I_680_ ratio remained stable between 24 and 36 h post injection (Fig. 5d), which provides a flexible window for hypoxia assessment. To further explore the proposed ratiometric imaging in ex vivo tissue, mice were euthanized after in vivo imaging and the tumors were dissected, split from the middle, and imaged. Representative images are shown in Fig. 5e. The I_800_/I_680_ image showed heterogeneous signal distribution within the tumor, representing the spatial distribution of hypoxia. To verify this ex vivo findings, hematoxylin and eosin (H&E) histopathology and immunofluorescence (IF) analysis of HIF-1α were performed to examine the tissue composition and hypoxia distribution. Representative pathology images of the tumor section outlined in Fig. 5e (white box) are presented. Indeed, the peripheral tissue region with higher I_800_/I_680_ ratio (solid arrows) correlated with the HIF-1α-positive area in the IF image (Fig. 5g, green). The central region with low I_800_/I_680_ and low HIF-1α expression was mainly the necrotic tissue. Therefore, ratiometric imaging of Cy7-1/PG5-Cy5@LWHA successfully highlighted the hypoxic tumors in vivo, and precisely delineated the spatial distribution of hypoxia in the ex vivo tissue.

### Quantitative imaging of tumors with different hypoxic levels

**Fig. 6:**
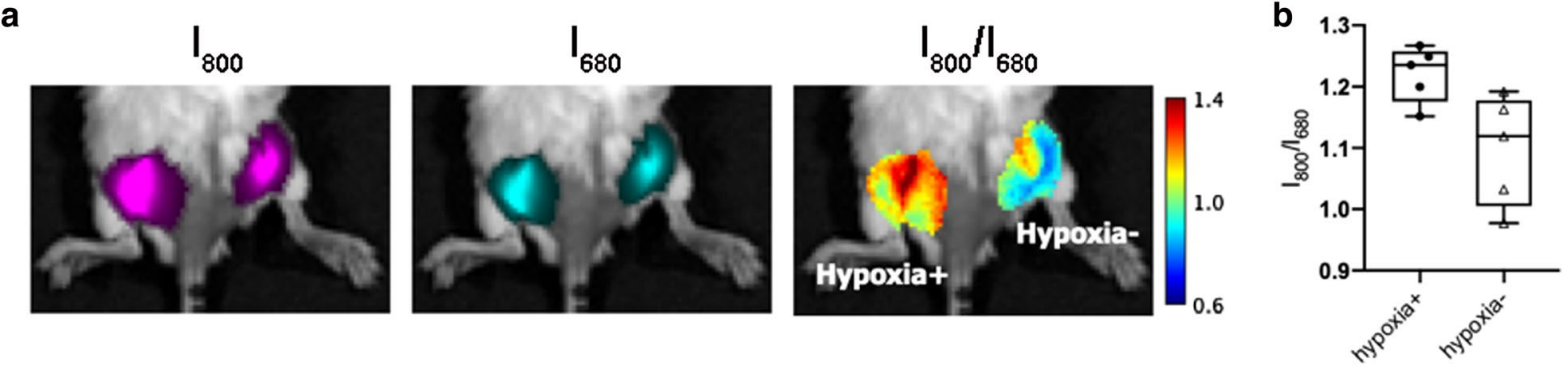
Nanoprobe response to the artificial tumor hypoxic environment. **a** Fluorescence and fluorescence ratio images of the artificial hypoxia^+^ and hypoxia^−^ microenvironments. Left tumor: hypoxia^+^, right tumor: hypoxia^−^. **b** Quantitative analysis of I_800_/I_680_ under different artificial microenvironments, error bars, mean $\pm$ s.d. (n = 5)

To explore whether the developed imaging approach could differentiate hypoxic and normoxic tumors in vivo, we again used CoCl_2_-treated 4T1 cells to create an artificial hypoxic microenvironment [26]. Cells cultured under regular conditions were used as normoxic controls. The same number of hypoxia^+^ and hypoxia^−^ cells (10^6^) were mixed with the nanoprobe (0.1 mg/ml, 100 µl) and injected into the left and right flanks of BALB/c mice. In vivo images were acquired 4 h after injection. As shown in Fig. 6a, the signal difference between hypoxia^+^ and hypoxia^−^ tumors was not evident in either Cy7-1 (I_800_) or Cy5 (I_680_) fluorescence images. However, in the I_800_/I_680_ ratio image, hypoxia^+^ tumor on the left exhibited a higher signal than the hypoxia^−^ control. Quantitative analysis of five mice demonstrated that the average I_800_/I_680_ ratio of hypoxia^+^ tumors was ~ 11% higher than that of the hypoxia^−^ tumors (Fig. 6b).

**Fig. 7:**
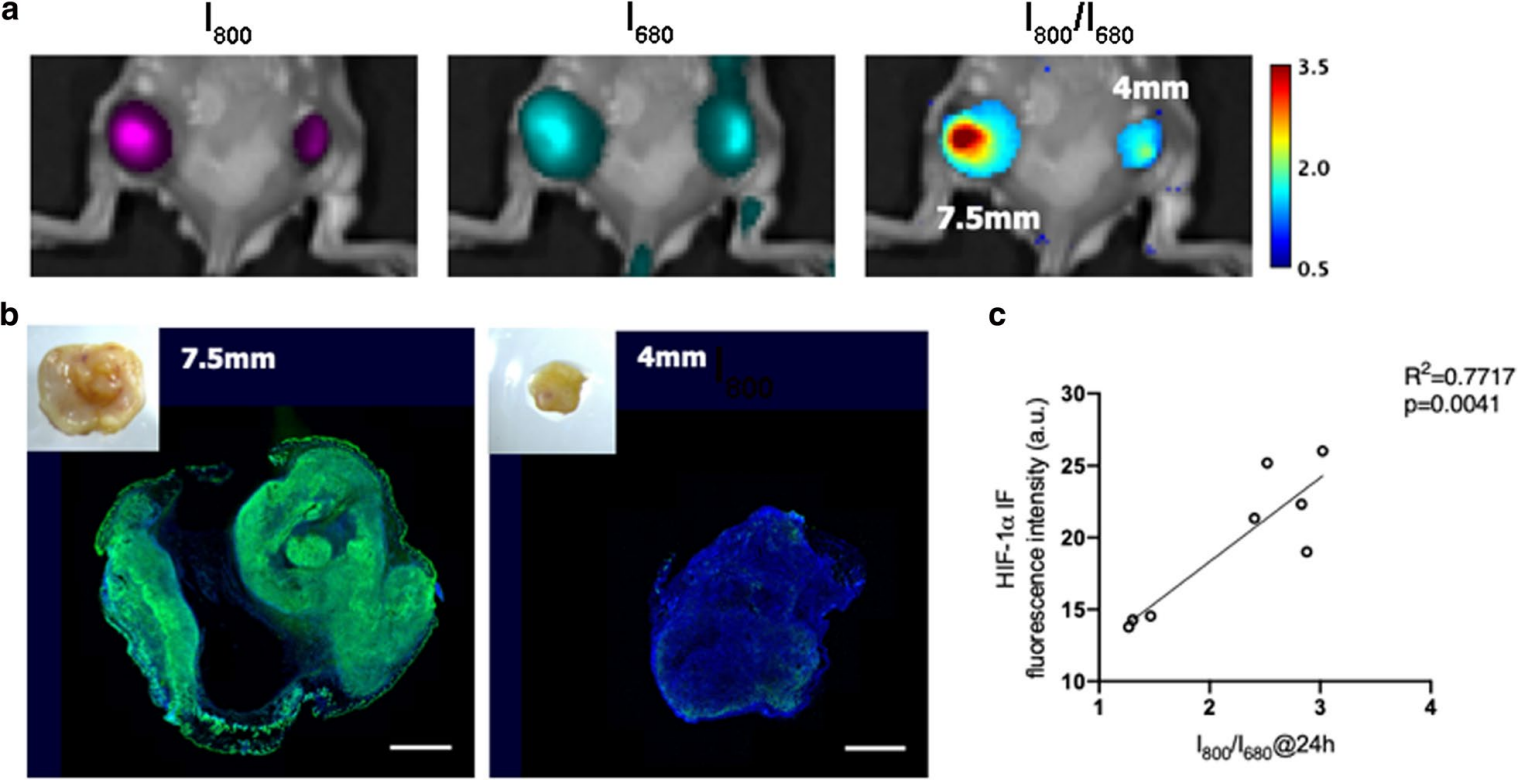
Imaging of tumors with naturally developed hypoxia. **a** Representative fluorescence and fluorescence ratio images of tumors with different sizes, left tumor: 7.5 mm, right tumor: 4 mm. **b** Digital photo and IF images of the tumors in **a**; green: anti-HIF-1 $\alpha$ staining, blue: DAPI, scale bar: 2 mm. **c** Scatter plot and correlation between HIF-1 $\alpha$ expression and in vivo I_800_/I_680_ ratio, simple linear regression, *p* = 0.0041, R^2^ = 0.7717, n = 8

To further validate the imaging strategy in a more clinically relevant setting, we established subcutaneous 4T1 tumors of different sizes on the bilateral flanks of BALB/c mice, representing a serial stage of naturally developed hypoxic tumors. We assumed that tumors with larger sizes would exhibit more severe hypoxia. For in vivo imaging, mice were intravenously injected with the nanoprobe and imaged with the IVIS system 24 h post injection. Similar to the results presented in Fig. 5, the fluorescence of both Cy5 (I_680_) and Cy7-1 (I_800_) could be observed in the tumor area, indicating the accumulation of the nanoprobe regardless of the tumor size. In the example shown in Fig. 7a, the left and right tumors exhibited comparable Cy5 fluorescence (I_680_), while the larger tumor (left flank, 7.5 mm) showed notably higher Cy7-1 fluorescence (I_800_) than the smaller tumor (right flank, 4 mm). In the I_800_/I_680_ ratio image, the signal from the 7.5 mm tumor appeared to be significantly higher than that from the 4 mm tumor, indicating a higher degree of hypoxia. The hypoxic status of the tumors was further confirmed by IF staining against HIF-1α. As shown in Fig. 7b, the 7.5 mm tumor exhibited much higher HIF-1 $\alpha$ expression than the 4 mm tumor, in consistent with the in vivo imaging result. Unlike the orthotopic tumors (Fig. 5), the subcutaneous tumors display a hypoxic gradient with stronger signal in the center of the tumor, which can be observed from both the in vivo ratio images and the ex vivo IF images. We believe that the different vasculature and microenvironment in different tumor models contribute to the distinctive hypoxia gradient. To further investigate the reliability of our imaging approach to quantify the hypoxic status, in vivo I_800_/I_680_ ratio was compared with the ex vivo IF result. As presented in Fig. 7c, HIF-1α expression level quantified from IF showed a significant correlation with the I_800_/I_680_ ratio from in vivo imaging, with R^2^ = 0.7717 and *p* < 0.005.

### Detection of lymph node metastasis using ratiometric hypoxia imaging

**Fig. 8:**
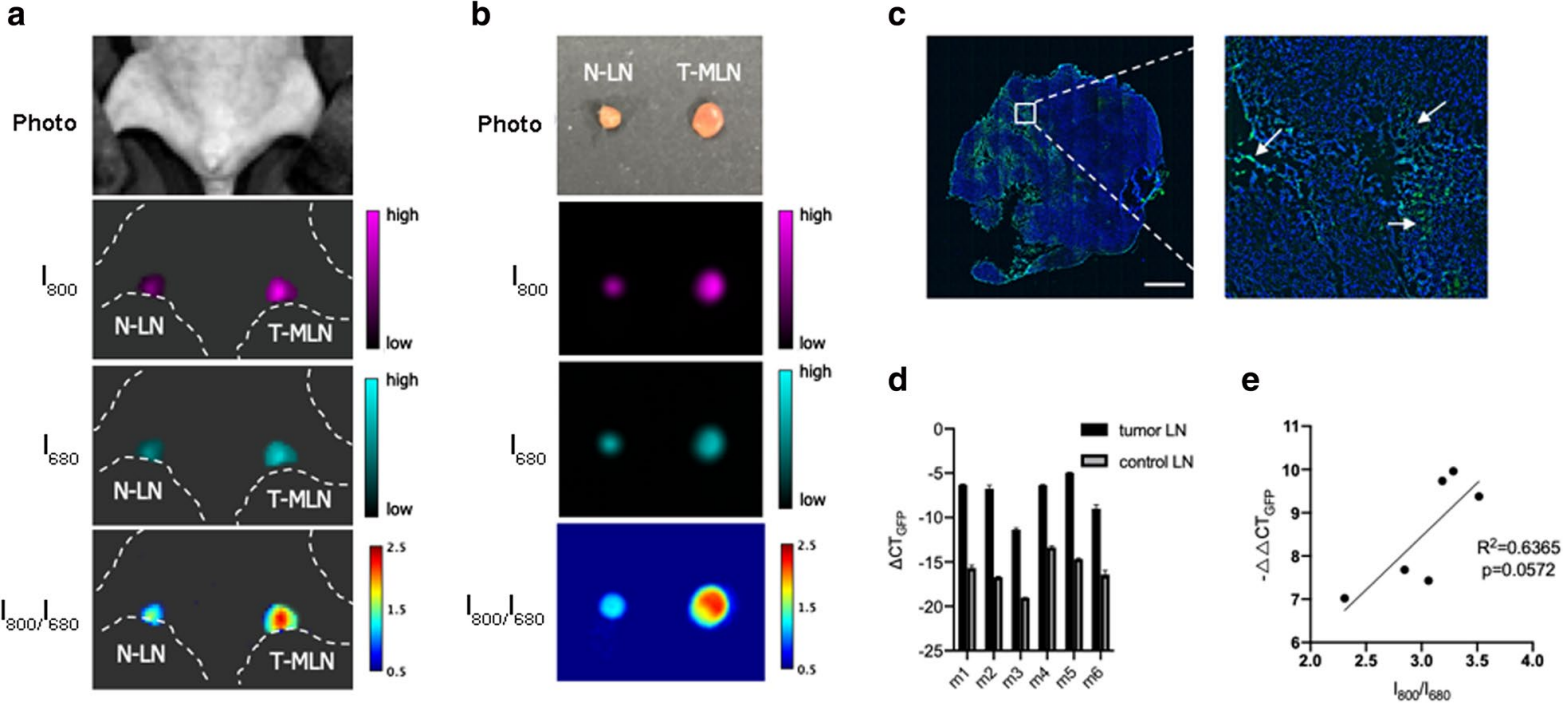
Imaging of lymph-node metastasis. **a** Digital photo, in vivo fluorescence and fluorescence ratio images of tumor-associated pLN and control pLN. **b** Digital photo, ex vivo fluorescence and fluorescence ratio images of the excised pLNs. **c** GFP fluorescence image confirming the presence of metastatic 4T1-GFP cells in the tumor-associated pLN; solid arrows: GFP fluorescence tumor cells. **d** qPCR showing the relative expression of GFP in the control and tumor-draining pLNs of each mouse, error bar, mean $\pm$ s.d. (n = 6). **e** Linear regression determined the correlation between in vivo imaging and qPCR results (R^2^ = 0.6365, *p* = 0.0572)

The microenvironment in the primary tumor can be very different from that in the metastatic site. Nevertheless, it has been discovered that the hypoxic profile of the primary tumor could be preserved in metastatic lesions [6]. Therefore, we hypothesized that elevated NTR activity would be a biomarker for detecting lymph node (LN) metastasis. To test our hypothesis, the LN metastasis mouse model was established by injecting 4T1-GFP breast cancer cells into the footpad of BALB/c nude mice. Three weeks after tumor inoculation, subcutaneous tumor can be observed in the footpad and enlarged popliteal LN (pLN) was palpable. For LN imaging, 1 mg/ml Cy7-1/PG5-Cy5@LWHA was injected intratumorally. The pLN on the opposite side was used as a control, and the same amount of nanoprobe was administered through footpad injection. Fluorescence images were acquired 3 h post injection. As shown in Fig. 8a, the nanoprobe was able to migrate to the pLNs, as revealed by the I_680_ fluorescence image. The tumor-associated pLN exhibited higher I_680_ signal intensity as compared to the control pLN, indicating more probe accumulation in the tumor-associated pLN. As a result, I_800_ or I_680_ image alone could not reveal the metastatic status of the node, since signal enhancement could either result from probe accumulation or elevated NTR activity. On the other hand, I_800_/I_680_ image rectified the concentration effect, highlighting the tumor-associated pLN. After imaging, LNs were excised and imaged under the same condition. Ex vivo imaging results were comparable to those of in vivo imaging, as shown in Fig. 8b. To verify the imaging findings, the LNs were cryosectioned and scanned with a fluorescence microscope. As shown in Fig. 8c, 4T1 cancer cells exhibiting GFP fluorescence (arrows) can be identified in the tumor-associated pLN, confirming the metastatic status. Furthermore, since the GFP gene is solely expressed by 4T1-GFP cells, GFP expression was determined using qPCR to evaluate the tumor burden in the nodes. The relative GFP expression was determined using the equation $\Delta CT={CT}_{GAPDH}-{CT}_{GFP}$, where ${CT}_{GAPDH}$ and ${CT}_{GFP}$ are the cycle threshold of the GADPH (internal control) and GFP genes, respectively. As shown in Fig. 8d, relative GFP expression in the metastatic LNs were distinctively higher than the control LN in all 6 mice. However, the GFP expression was slightly different among individual mice, suggesting different tumor burden in the metastatic nodes. Since NTR activity is an endogenous property of tumor cells, we further investigated whether I_800_/I_680_ ratio of Cy7-1/PG5-Cy5@LWHA could reflect the amount of tumor cells in the LNs. For each mouse, tumor burden was determined using the equation $\Delta \Delta CT={\Delta CT}_{T-MLN}-{\Delta CT}_{N-LN}$, where ${\Delta CT}_{T-MLN}$ and ${\Delta CT}_{N-LN}$ are the cycle threshold of the tumor metastatic LN and normal LN, respectively. As shown in Fig. 8e, in vivo I_800_/I_680_ showed a good linear correlation with the tumor burden in the metastatic nodes, with R^2^ = 0.6365 and *p* = 0.0572. To this end, we demonstrated the feasibility of using ratiometric imaging of Cy7-1/PG5-Cy5@LWHA to identify metastatic LNs in vivo. Quantitative imaging also allowed for tumor burden estimation with accuracy comparable to that of qPCR.

## Discussion

Activatable molecular probes have gained significant interest in recent years because of their superior sensitivity and specificity (in comparison with traditional “always-on” probes) [27]. Small-molecule probes that can be activated by hypoxia, pH, ROS, and enzymes have been developed for tumor imaging [13–17, 28–31]. However, the in vivo use of activatable probes with single fluorescence emission remains challenging. Although most of the activatable probes were delicately designed to exhibit linear signal dependency on the concentration of the targeted analytes, few were able to provide quantitative information reliably in vivo.

This problem is especially prominent in hypoxia imaging, as regions with severe hypoxia are often affected by higher levels of vascular abnormalities and limited probe delivery [18]. In this study, we developed a dual-emission activatable nanoprobe, Cy7-1/PG5-Cy5@LWHA, which allows for self-calibration and quantitative hypoxia imaging. Our results show that the proposed imaging approach can accurately differentiate tumors with different hypoxic levels, which provides a novel imaging technique for evaluating hypoxia in vivo. This ratiometric approach and dual-emission probe design can be easily used with different applications by simply replacing Cy7-1 with another activatable dye. In this study, Cy5 was used as the “always-on” fluorophore because its fluorescence spectrum can be easily separated from that of Cy7-1. Future studies may introduce NIR-II fluorophores as an internal reference with lower interference in tissue scattering [32].

In addition, our results showed that hypoxia imaging successfully identified the metastatic LNs. Previous studies have shown that the hypoxia signatures of the primary tumor may be preserved at the metastatic site [6]. Our results suggest that elevated NTR activity is a hypoxia signature that could be used as an imaging target for LN metastasis. A common problem in LN mapping is that tumor growth in the LNs alters the interstitial pressure and lymph flow rates, leading to heterogeneous probe delivery. As a result, the metastatic status of the LN could not be reliably determined using single-emission probes. To address this problem, previous studies have used a dual tracer approach, simultaneously using targeted and untargeted probes, to eliminate the concentration effect [33]. However, the two tracers need to have identical pharmacokinetics but different target affinities, which is very demanding in terms of probe design and synthesis, as both probes need to have the same molecular weight, size, and zeta potential. In our system, we coupled hypoxia-activatable and “always-on” fluorophores using a nanocarrier, to ensure identical pharmacokinetics. The “always-on” Cy5 fluorescence helped to track the LNs in the tumor region, and the ratio between the hypoxia-activatable signal and the “always-on” signal enabled quantitative identification of the metastatic nodes. This information could potentially assist clinical decision-making during surgery to locate and diagnose metastatic LNs in real time.

## Conclusion

Overall, this study developed a novel imaging strategy combining a self-calibrated activatable nanoprobe with ratiometric image analysis, for the quantitative assessment of tumor hypoxia in vivo. Using this strategy, dynamic changes of hypoxia during tumor growth could be monitored in vivo in real time. The successful detection of metastatic LNs presented in this work further validated the potential clinical translation of this imaging strategy.

## Materials and methods

### Probe Synthesis and Characterization

Cy5-NHS (146368-14-1, RuixiBio, Shaanxi, China) was first labeled with PG5 (536709, Sigma-Aldrich, St. Loius MA, USA) to form PG5-Cy5. Briefly, 300 mg of PG5 was dissolved in 5 ml of borate buffer (pH 9.0). To this solution, Cy5-NHS (3 mg) prepared in DMF was added and stirred for 24 h at room temperature in the dark. The reaction product was then transferred into a 5 K MWCO dialysis bag (YA1058, Solarbio, Beijing, China) and dialyzed against ddH_2_O for two days. Purified PG5-Cy5 was retrieved by freeze-drying. Cy7-1 was synthesized in accordance with a previously reported protocol [18]. To encapsulate Cy7-1 in the interior of PG5-Cy5, 2 mg Cy7-1 was dissolved in acetone (200 ${\mu}\mathrm{l}$) and slowly added to PG5-Cy5 (4 mg) in ddH_2_O (8 ml). After stirring on ice for 2 h, the mixture was transferred to a 10 kDa ultrafiltration tube (UFC801096, Millipore, Burlington, MA, USA) and centrifuged at 5500 rpm for 15 min at 4 °C. Subsequently, Cy7-1/PG5-Cy5 was functionalized with LWHA (R-HC-5 K, RuixiBio, Shaanxi, China). Cy7-1/PG5-Cy5 was mixed with LWHA at a mass ratio of 1:1 in ddH_2_O. The mixture was vortexed for 30 s and incubated at 4 °C for 1 h. Finally, the reaction product was dialyzed against ddH_2_O overnight to obtain purified Cy7-1/PG5-Cy5@LWHA. To confirm the successful synthesis of the nanoprobe, absorption and fluorescence spectra were measured after each synthesis step. Absorption spectra were measured on a spectrophotometer (UV-3600Plus, Shimadzu, Kyoto, Japan), and fluorescence spectra were measured using a fluorometer (F-7000, Hitachi, Tokyo, Japan). The hydrodynamic size and zeta potential were measured using a Dynamic Light Scattering Zetasizer (Nano-ZS90, Malvern Instruments, Malvern, UK). The size and morphology of the nanoprobes were observed by TEM (JEM 1200EX, Jeol, Tokyo, Japan). The composition of the nanoprobe was characterized by Fourier transform infrared spectroscopy (Nicolet iS10, Thermo Fisher Scientific, Waltham, MA, USA).

### Probe response to NTR in vitro

To verify the probe’s response to NTR, 0.25 mg/ml Cy7-1/PG5-Cy5@LWHA in Tris buffer was mixed with 500 $\upmu \text{M}$ NADH (N8129, Sigma-Aldrich, St. Louis, MA, USA) and 0.5 $\upmu {\text{g/ml}}$ NTR (N9284, Sigma-Aldrich, St. Louis, MA, USA). The solution was incubated at 37 ºC for 10 min. The fluorescence spectra of the nanoprobes were recorded before and after the reaction. To show the ratiometric imaging performance of the probe, Cy7-1/PG5-Cy5@LWHA, NADH, and NTR were mixed in 96-well plates, incubated at 37 °C for 10 min, and imaged using an IVIS imaging system (PerkinElmer, Waltham, MA, USA). Nanoprobe concentrations were modulated from 0 to 0.29 mg/ml and NTR concentrations ranging from 0 to 1.5 μg/ml were used. Cy5 was excited at 640 nm and emission was recorded at 680 nm. Cy7-1 was excited at 745 nm and emission was recorded at 800 nm. Regions of interest (ROIs) were selected to include the area of each well. The mean fluorescence intensities of Cy7-1 (I_800_) and Cy5 (I_680_) were generated for each ROI in ImageJ. The I_800_/I_680_ ratio was calculated and the I_800_/I_680_ vs. NTR concentration curve was plotted for each probe concentration.

### Cell culture and in vitro imaging

Mouse breast cancer cell lines 4T1 and 4T1 transfected with Green fluorescence protein (4T1-GFP) genes were grown in RPMI-1640 medium (Gibco, Waltham, MA, USA) supplemented with 10% fetal bovine serum (Gibco, Waltham, MA, USA). Cells were cultured in a humidified atmosphere containing 5% CO_2_ at 37 ºC. To induce hypoxia in cell culture, 4T1-GFP cells were incubated with 70 µM CoCl_2_ (Macklin, Shanghai, China) in serum-free RPMI medium overnight. For confocal imaging, cells were seeded at a density of 50,000 cells in 35 mm glass-bottomed imaging dishes (Biosharp, Hefei, China) and cultured overnight. Prior to imaging, cells were incubated with the nanoprobe (0.05 mg/ml) for 4 h and washed three times with PBS (Gibco, Waltham, MA, USA). Cells were then incubated with 2 μg/ml DAPI (Solarbio, Beijing, China) for 20 min to label the nuclei. To block CD44, cells were first incubated with 0.05 mg/ml LWHA for 2 h. In vitro imaging was performed using a confocal microscope (Andor Dragonfly, Oxford Instruments, Abingdon, UK) with a 40 $\times$ objective. For the Cy5 and Cy7-1 channels, cells were excited at 637 nm and 730 nm, respectively.

### Cytotoxicity test

Cytotoxicity was evaluated using the MTT cell proliferation and cytotoxicity assay kit (M1020, Solarbio). 4T1 cells were seeded in 96-well plates and cultured overnight in a humidified atmosphere containing 5% CO_2_ at 37 ºC. After monolayer formation, different concentrations of the nanoprobe (0, 1, 2, 10, 50 and 100 $\mathrm{\mu g}/\mathrm{ml}$) were added to the cells and incubated for 4 h. After incubation, the cells were washed with PBS and 90 $\mathrm{\mu l}$ RPMI-1640 medium and 10 $\mathrm{\mu l}$ MTT solution were added to each well and incubated with the cells for another 4 h. Afterward, the supernatant was discarded and 110 $\mathrm{\mu l}$ formazan was added to each well. The plates were gently shaken for 10 min. The OD_490_ of each well was measured using a microplate reader (Biotek, Vinooski, VT, USA).

### In vivo imaging of orthotopic tumor model

Following ethical approval No. IA-202105, all animal experiments were performed in compliance with the guidelines set by the Animal Care Committee at the Institute of Automation, Chinese Academy of Science (license #SYXK(京) 2020–0049). To establish the orthotopic breast tumor model, 4T1 cells (1 $\times$ 10^6^ cells in 100 µ $\mathrm{l}$ PBS) were injected into the 4^th^ inguinal mammary fat pad of female BALB/c mice (6 weeks old, Charles River). Tumor growth was monitored for 3–4 weeks post inoculation. Once the tumor size reached 5–10 mm, 200 μl Cy7-1/PG5-Cy5@LWHA nanoprobe (1 mg/ml in PBS) was injected into the mice via the tail vein. Mice were imaged at 4, 8, 12, 24, 28, 32, 36, and 48 h post injection using the IVIS imaging system. The image settings for Cy5 and Cy7-1 were the same as those described above. At 48 h post injection, the mice were sacrificed, and the tumor and major organs were collected and imaged using an IVIS imaging system.

### In vivo imaging of artificial hypoxic microenvironment

To establish an artificial hypoxic tumor microenvironment, 4T1 cells were incubated with 70 $\upmu \text{M}$ CoCl_2_ in serum-free RPMI medium overnight. Hypoxic cells were collected and resuspended in PBS at a density of 1 $\times$ 10^7^ cells/ml. Hundred microliter of cell suspension was mixed with 100 $\mathrm{\mu l}$ Cy7-1/PG5-Cy5@LWHA nanoprobe (0.1 mg/ml) and subcutaneously injected into the right flank of female BALB/c mice. As a normoxic control, the same number of 4T1 cells cultured under regular conditions were mixed with Cy7-1/PG5-Cy5@LWHA and injected into the left flank of the same mouse. Mice were imaged with an IVIS imaging system 4 h post injection.

### In vivo imaging of tumors with different hypoxic status

To investigate the ability of the nanoprobe to quantify tumor hypoxic status, subcutaneous 4T1 tumors of different sizes were established on the flanks of female BALB/c mice. In each mouse, two tumors were grown on the bilateral flanks. The first tumor was implanted on the left flank by subcutaneous injection of 1 $\times$ 10^6^ 4T1 cells. Once it reached a palpable size at 10 days post inoculation, a second tumor was similarly established in the right flank. Ten days after the second inoculation, two tumors of different sizes were established. For in vivo imaging, mice were intravenously injected with 200 μl of Cy7-1/PG5-Cy5@LWHA (1 mg/ml) and imaged with the IVIS imaging system 24 h post injection.

### In vivo imaging of metastatic LN

To establish LN metastasis of 4T1 breast cancer cells, 1 $\times$ 10^5^ 4T1-GFP cells resuspended in 30 $\mathrm{\mu l}$ PBS were injected into the left footpad of Balb/c nude mice. Tumor growth was monitored for 3 weeks until a palpable pLN was observed. For in vivo imaging, 30 µ $\mathrm{l of}$ Cy7-1/PG5-Cy5@LWHA nanoprobe (1 mg/ml) was injected intratumorally into the left footpad. The same amount of nanoprobe was injected into the right footpad as a control. In vivo images of LN were acquired using the IVIS imaging system at 3 h post injection.

### Ratiometric processing and hypoxia quantification

Pixel-based ratio images were generated in ImageJ 1.53a according to the following equation:

$${\frac{{{I_{800}}}}{{{I_{680}}}}_{i,j}} = \left\{ \begin{array}{l} NAN,\;if\;{I_{800}}_{i,j} < p\;OR\; \in {I_{680}}_{i,j} < q,\\ {\frac{{{I_{800}}}}{{{I_{680}}}}_{i,j}}if\;{I_{800}}_{i,j} \ge p\;AND\;{I_{680}}_{i,j} \ge q \end{array} \right.$$

where i and j denote the pixel coordinates, $I_{800}$ was the pixel value of the Cy7-1 fluorescence image, $I_{680}$ is the pixel value of the Cy5 fluorescence image, $p$ is the threshold value chosen to remove the background in the Cy7-1 fluorescence image, $q$ is the threshold value chosen to remove the background in the Cy5 fluorescence image. To obtain the ratio information of a certain area, the ROI was selected, and the average value $\frac{{I_{800} }}{{I_{680} }}_{ave}$ was calculated. For all computations, images were converted to 32-bit and processed.

### Histological evaluation

Excised tissue was fixed in 10% formalin and embedded in paraffin; 4 μm-thick sections were sectioned with a microtome and stained with H&E. Immunofluorescence was used to evaluate HIF-1α expression. The antibodies and staining reagents used were anti-HIF-1α (ab228649, Abcam, Cambridge, UK), 488-conjugated goat anti-rabbit IgG (GB25303, Servicebio, Wuhan, China), and DAPI (G1020, Servicebio, Wuhan, China). Immunofluorescence was performed according to an established protocol (www.abcam.com/protocols). H&E and fluorescence images were acquired using a Pannoramic MIDI scanner (3DHISTECH).

### qPCR

*GFP* expression was analyzed to evaluate the tumor burden in metastatic LNs. Briefly, LNs were excised and freshly frozen in RNAwait reagent (SR0020, Solarbio, Beijing, China). Total RNA was isolated according to the protocol provided in www.abcam.com/protocols. To assess gene expression, cDNA was prepared using the Servicebio ®RT First Strand cDNA Synthesis Kit (G3330, Servicebio, Wuhan, China). Fifteen microliter reaction was set up using 7.5 ${\mu}\mathrm{l}$ 2× SYBR Green qPCR Master Mix (G3320, Servicebio, Wuhan, China), 2.5 $\upmu \text{M}$ primer forward (GFP: GGGACCGCTCCTTCCTGTT, GAPDH: CCTCGTCCCGTAGACAAAATG) and primer reverse (GFP: ACGGGGATGATCTTCTCGCA, GAPDH: TGAGGTCAATGAAGGGGTCGT), 2.0 ${\upmu }$ l cDNA template and 4 µl ddH_2_O. qPCR was performed using the CFX96 Touch Real-Time PCR Detection System (Bio-Rad, Hercules, CA, USA). Each sample was tested in triplicate. GAPDH was used as an endogenous control. The relative GFP gene expression level was determined using $\Delta CT = CT_{GAPDH} - CT_{GFP}$, where CT is the threshold cycle. The tumor burden in the metastatic LNs was represented by $\Delta \Delta CT = \Delta CT_{T - MLN} - \Delta CT_{N - LN}$.

### Statistical analysis

Data analysis was performed using GraphPad Prism ver. 9. For in vitro imaging experiments, an unpaired *t-*test with Welch's correction was used to determine the statistical difference. For in vivo imaging experiments, the Wilcoxon matched-pairs signed rank test was used to determine the differences between different hypoxia groups. Simple linear regression was used to compare ex vivo HIF-1 $\alpha$ expression with in vivo I_800_/I_680_ at 24 h, and to compare tumor burden determined from $\Delta \Delta CT$ of qPCR with in vivo I_800_/I_680_ in tumor-draining LNs. All data are presented as mean ± s.d. Statistical significance was set at *p* < 0.05.

## Supplementary Information

**Additional file 1: Figure S1**. FTIR spectrum of Cy7/PG5-Cy5@LWHA.

**Additional file 2: Figure S2**. NTR response of free Cy7-1.

**Additional file 3: Figure S3**. Cytotoxicity of (a) Cy7-1/PG5-Cy5@LWHA and (b) Cy7-1/PG5-Cy5.

## Data Availability

All data required to reproduce the results can be obtained from the corresponding author upon request.

## Abbreviations

CT: Cycle threshold
HIF-1: Hypoxia-inducible transcription factor
H&E: Hematoxylin and eosin
IF: Immunofluorescence
LWHA: Low molecular weight hyaluronic acid
LN: Lymph node
NTR: Nitroreductase
PG5: Poly(amidoamine) dendrimer, 5th generation
TEM: Transmission electron microscopy
