# Supplementary figures and images for: Quantitative hypoxia mapping using a self-calibrated activatable nanoprobe
Source: J Nanobiotechnology. 2022 Mar 18;20:142. doi: 10.1186/s12951-022-01341-9 (PMC8931977; doi:10.1186/s12951-022-01341-9)

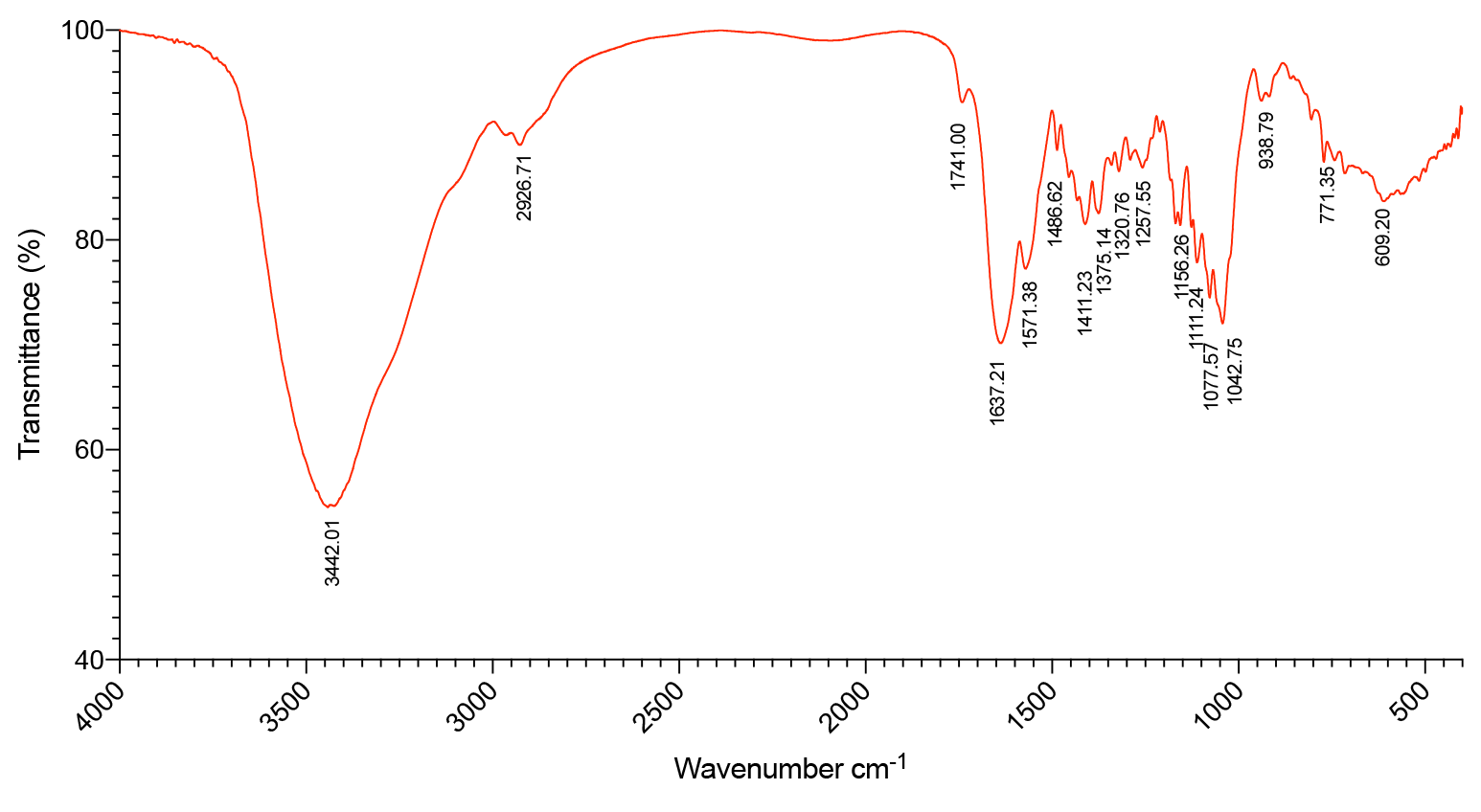

Supplement: Supplementary file 1 — Additional file 1: Figure S1. FTIR spectrum of Cy7/PG5-Cy5@LWHA. [file 12951_2022_1341_MOESM1_ESM.tif]

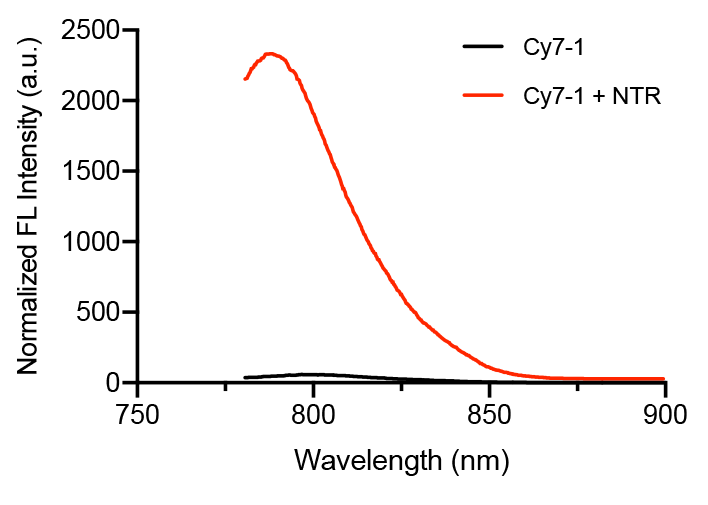

Supplement: Supplementary file 2 — Additional file 2: Figure S2. NTR response of free Cy7-1. [file 12951_2022_1341_MOESM2_ESM.tif]

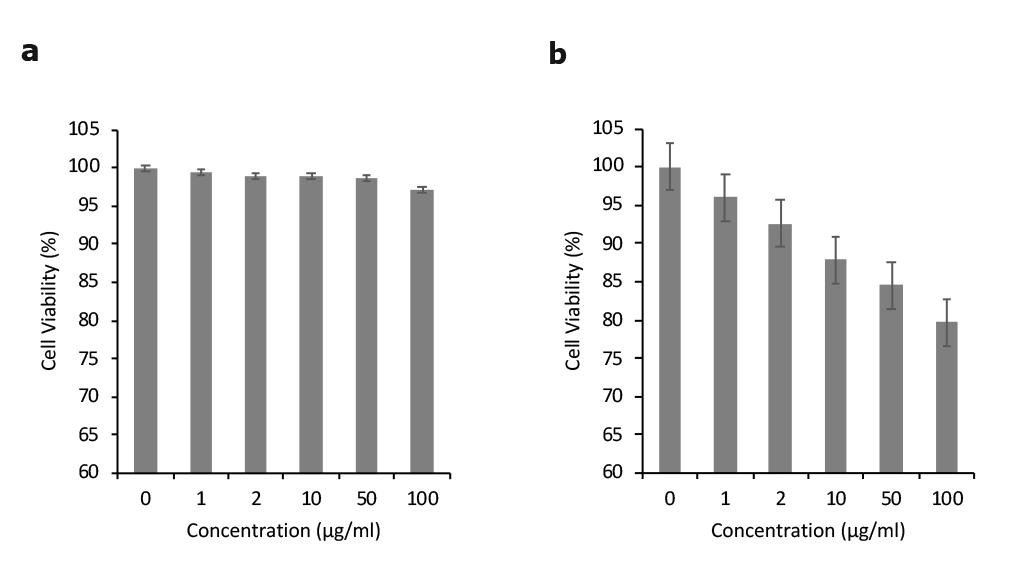

Supplement: Supplementary file 3 — Additional file 3: Figure S3. Cytotoxicity of (a) Cy7-1/PG5-Cy5@LWHA and (b) Cy7-1/PG5-Cy5. [file 12951_2022_1341_MOESM3_ESM.tif]
